# Optimizing Metasurface-Component Performance by Improving Transmittance and Phase Match of the Nanopillars

**DOI:** 10.3390/nano12213720

**Published:** 2022-10-23

**Authors:** Xiaohong Sun, Shuang Huo, He Yang, Mengmeng Yan, Jianing Zhai, Saili Zhao, Yong Zeng

**Affiliations:** Henan Key Laboratory of Laser and Optoelectronic Information Technology, The School of Electrical and Information Engineering, Zhengzhou University, Zhengzhou 450001, China

**Keywords:** metasurface, metalens, phase correction, spherical aberration

## Abstract

In the propagation phase of a dielectric metasurface, there are two important problems. Firstly, the range of transmittance of the nanopillars for a building metasurface is usually between 60% and 100%, which reduces the metasurface’s overall transmittance and affects the uniformity of the transmitted light. Secondly, the realistic phase provided by the nanopillar cannot be matched very well with the theoretical phase at each lattice location.The phase difference (between a realistic phase and theoretical phase) may reach tens of degrees. Here, we propose an interesting method to solve these problems. With this new method, a metalens is designed in this paper. The nanopillars for building the metalens have transmittance over 0.95, which increases the metalens transmittance and improves the light uniformity. In addition, with the new method, the phase differences of all elements in the metalens can also be reduced to be below 0.05°, decreasing the metalens spherical aberration dramatically. This method not only helps us to optimize the metalens but also provides a useful way for designing high-quality metasurfaces.

## 1. Introduction

Unlike classical convex microlenses based on glass refraction [1,2,3], the metasurface has nanoscale size, better imaging quality and flexible interfacial phase modulation capability. These characteristics make the metasurfaces have great information capacity and can be highly integrated in future optoelectronic systems. Due to these advantages and its potential perspectives, metasurface technology has been an intriguing topic in recent years [4,5,6,7,8,9,10,11,12,13,14,15,16,17]. The metasurfaces usually consist of subwavelength nanoantenna arrays. The arrays are used to precisely tailor the amplitude, phase, and polarization of light. Based on these optical responses, many metasurfaces applications can be developed. A lot of achievements have been obtained in the metasurface study, such as beam deflection [18,19,20,21,22], holographic imaging [23,24,25,26,27], vortex beams [28,29,30], polarization conversion [31,32,33] and so on. In the numerous metasurface research directions, one kind is to control the light wavefront based on the nanopillar transmission phase [34,35,36,37,38,39]. In this direction, the propagation phase should be equal to the desired value calculated with the element position and design metasurface parameters. By controlling the geometrical parameters of the nanopillars, the propagation phase modulation can cover the 2π full range and then fully manipulate the light properties.

However, there are two problems in the actual design for the propagation-phase metasurfaces. One of them is that the range of transmittance of the nanopillars for building a metasurface is usually between 60% and 100%, and the low of transmittance of the nanopillar unit reduces the overall transmittance of the metasurface. Building a metasurface component requires a large number of nanopillars of different sizes. Since the light traveling through nanopillars is in a resonant manner, the different sizes of nanopillars will make their transmittance different. Hence, the nanopillar transmittance usually covers a wide range. This phenomenon will reduce the metasurface transmittance and affect the uniformity of its transmission light. To solve this problem, the metasurface is usually constructed by the nanopillars with transmittance above a specific threshold, abandoning the nanopillars with low transmittance [40,41,42,43,44,45,46,47,48]. In this way, the problem has been somewhat improved. Nevertheless, the thresholds in these works were 0.6, 0.7, 0.85, 0.9, 0.94, and so on, which can be further improved. In addition, this method abandons a lot of nanopillars, which means that the number of phases that the nanopillars can modulate is dramatically reduced. Thus, the approach will further amplify the phase difference of the next problem (see later for details).

The second problem is that the desired phase (DP) calculated with the designed parameters (e.g., the position coordinates and focal length) cannot be matched very well with the realistic phase modulated by the nanopillar. The phase difference (between the realistic phase and desired phase at each lattice location) may reach tens of degrees. As we know, the modulation phase is discretized in the actual design, which means that the propagation phase cannot contain all values from 0 to 2π. On the other hand, the metasurface meta-atoms are regularly distributed, which results in a fixed location for each element. In this way, calculated with the position and design metasurface parameters, the desired phase shift induced by an element becomes constant. Thus, the desired constant phase of an element should be matched with a value selected from the discretized propagation phase. In this case, there must be a difference between them. Usually, the difference is not negligible and will affect the metasurface properties. Although it is significant for metasurface research to reduce the phase difference, there is little discussion of this problem. Xie et al. produced a perfect vortex beam using a propagation phase dielectric metasurface [49]. In this paper, the difference between the actual and theoretical phase was explicitly mentioned to be controlled within 3.5°.

In this paper, we firstly propose and demonstrate an optimization method to solve these problems. First of all, to further improve the first problem, the threshold transmittance of the nanopillars is set to be 0.95 in this work. To our knowledge, this threshold value is the largest one compared with that of published works [40,41,42,43,44,45,46,47,48]. This enables the metasurface to have higher transmittance and more uniform light field distribution. Secondly, we propose and use a method to correct the difference between the DP and the nanopillar propagation phase. As we know, the DP of an element is calculated with the position and metasurface parameters. Thus, we can finely adjust the DP by slightly changing the position. Then, the DP can be matched very well with the propagation phase by selecting an appropriate nanopillar. We have developed a process for making such an optimized metalens and analyzed the effect of phase difference on the metalens. The analysis shows that the metalens performance is improved dramatically with this method, including the meta-atom phase differences, transmittance, and the metalens spherical aberration. This work should provide a new method to design and fabricate a metasurface with greater properties, and the method can be widely used in the metasurface field.

## 2. The Optimization Methods of Nanopillars

In this section, we will demonstrate the proposed method in detail. In a nutshell, our method is to further increase the transmittance threshold and sharply decrease the phase difference. We will increase the threshold in a common way, by specifying that all used nanopillars should have a transmittance of over 0.95. In contrast, we will decrease the phase difference by finely adjusting the placement position of the nanopillar.

**Figure 1:**
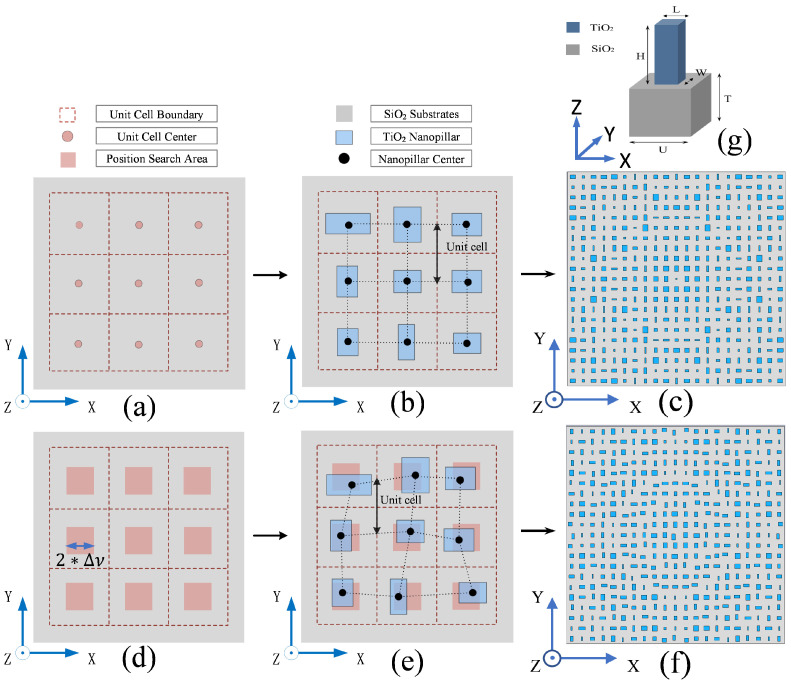
(Color online) The comparison schematics between the traditional and newly proposed methods. (**a**) The position distribution to build nanopillars in the traditional method. (**b**) The traditional method for designing a metasurface. The nanopillar transmittance is in a wide range, and the nanopillar placement is fixed and regular. (**c**) A traditional metalens designed with the traditional method. The nanopillars are regularly distributed. (**d**) The search area distribution to build meta-atoms in the newly proposed method. (**e**) The newly proposed method for designing a metasurface. The nanopillar transmittance is high and uniform, and the nanopillar arrangement is irregular. (**f**) The position modified metalens intended with the new approach. (**g**) The stereogram of the unit cell, where U = 400 nm, T = 400 nm, H = 500 nm, and L,W∈[50,250] nm with a step of 5 nm.

In the usual metasurface design, by changing the length and width, many nanopillars are simulated, and the relevant data are obtained. Then, a database table is established with the simulated results. In this table, every nanopillar with a certain length and width has a corresponding transmittance and propagation phase. Based on the database table, a metasurface component can be designed as follows. Firstly, a substrate is established, and its surface is divided into a number of unit cells. Then, the desired shift phase at every unit cell center is calculated. Taking a metalens design as an example, the desired phase $\varphi_{DP}\left(x_{c},y_{c}\right)$ should satisfy the following equation.

(1) $$\varphi_{DP}\left(x_{c},y_{c}\right)=\frac{2\pi }{\lambda }\left(\sqrt{{x_{c}}^{2}+{y_{c}}^{2}+f^{2}}-f\right).$$

where *f*, λ are the metalens focal length and the light wavelength, respectively, and $\left(x_{c},y_{c}\right)$ are the two-dimensional coordinates of a unit cell center. Next, compared with the calculated desired value, the nearest propagation phase (NPP, represented as $\varphi_{NPP}\left(x_{c},y_{c}\right)$ ) is selected from the database table. In the end, the nanopillar corresponding to the selected $\varphi_{NPP}\left(x_{c},y_{c}\right)$ is put on the substrate surface at the position $\left(x_{c},y_{c}\right)$. In this way, the metasurface component can be established (details seen in Figure 1a,b). The above process is the common way to design a metasurface component. In this method, the transmittance recorded in the database is in a wide range (almost full 0–1 range in this paper), and the recorded propagation phases are discrete and finite. The wide-range transmittance damages the overall metasurface transmittance and the light uniformity. Moreover, the DP at a unit cell center is constant because of the fixed $x_{c}$, $y_{c}$, *f* and λ. Thus, there must be a non-negligible difference between the DP and the recorded discrete phases at many unit cell locations.

Different from the ordinary way, before the database table is established with the simulated results, the data with a transmittance of below 0.95 are deleted. Therefore, the nanopillars used to build the metasurface have higher and more uniform transmittance. On the other hand, the placement position of the nanopillar is no longer fixed at the unit cell center here. As shown in Figure 1d,e, a position search area is delineated in the middle of each unit, and the nanopillar position is finely tunable in this area. In our method, during the metasurface component design, the phase difference ($\Delta \varphi_{c,c}=\left|\varphi_{NPP}\left(x_{c},y_{c}\right)-\varphi_{DP}\left(x_{c},y_{c}\right)\right|$) at the unit center is calculated firstly. If $\Delta \varphi_{c,c}<0.05$°, the corresponding nanopillar will be built at the unit cell center. Otherwise, the nanopillar position will be finely adjusted in the delineated area to reduce the phase difference. The detailed operations are shown as follows. (I) The delineated position search area is divided into n×n regions (e.g., *n* = 30). (II) The coordinates of the regions are defined as $\left(x_{i},y_{j}\right)$, as shown in Equations (2) and (3). In the equations, i,j∈(1,n) are integers, and Δv is a parameter to demarcate the border of the delineated area. Considering the actual manufacturing resolution, and avoiding overlap of the nanopillars, the Δv should be set at an appropriate value. (III) After setting the *i* and *j* values, the phase difference $\Delta \varphi_{i,j}=\left|\varphi_{NPP}\left(x_{i},y_{j}\right)-\varphi_{DP}\left(x_{i},y_{j}\right)\right|$ is calculated. If $\Delta \varphi_{i,j}<0.05$°, the corresponding nanopillar will be built at the position $\left(x_{i},y_{j}\right)$. Otherwise, *i* or *j* will be changed with a step of 1, and the III process is repeated. (IV) In case none of the n×n regions satisfy Δφ<0.05°, one of the following actions will be performed. (i) Repeat the above steps after increasing Δv. (ii) Repeat the above steps after increasing *n*. (iii) The region with the minimum $\Delta \varphi_{i,j}$ will be selected, and the corresponding nanopillar will be built at this position. These four processes will be repeated at every unit to reduce the phase difference.

(2) $$x_{i}=\left(x_{c}-\Delta v\right)+2\Delta v\frac{\left(i-1\right)}{\left(n-1\right)}$$

(3) $$y_{j}=\left(y_{c}-\Delta v\right)+2\Delta v\frac{\left(j-1\right)}{\left(n-1\right)}$$

## 3. The Optimization Results

With the newly proposed method, we develop the design process of an optimized metalens and analyze the performances of the metalens. In our design, the primary unit cell consists of a SiO${}_{2}$ substrate and a high-aspect-ratio TiO${}_{2}$ nanofin, as shown in Figure 1g. The length and width of the SiO${}_{2}$ brick are U, representing the lattice constant of the meta-atoms array. The SiO${}_{2}$ substrate has a thickness of *T*. In addition, the structure parameters of the TiO${}_{2}$ nanopillar are *L*, *W* and *H*. By changing *L* and *W*, the propagation phase modulation can cover the 0−2π full range. With this unit cell, a typical metalens and an optimized metalens are designed. The metalenses contain 21 × 21 unit cells and have a focal length of 6000 nm for the 532 nm light. As shown in Figure 1c, a traditional dielectric metalens is built by the traditional method. For the traditional method, we pick a regular array of subwavelength-spaced locations for the nanopillars, find the desired phase at each location, and then use the results of simulations to obtain the best nanopillar for creating that phase. However, in this metalens, the nanopillar transmittance threshold is not improved, and the phase difference is not corrected. As shown in Figure 1a, the TiO${}_{2}$ nanopillars are arranged in regular arrays on the substrate. In the meantime, an optimized metalens is designed with the new method, as shown in Figure 1f. In the optimized metalens, the transmittance of all nanopillars is improved to be over 0.95, and the phase difference of that is corrected to be below 0.05°. It is evident that the positions of some nanopillars were fine-tuned on the basis of the periodic distribution. These nanopillars are put away from the unit cell center for phase correction.

### 3.1. The Threshold Improvement

**Figure 2:**
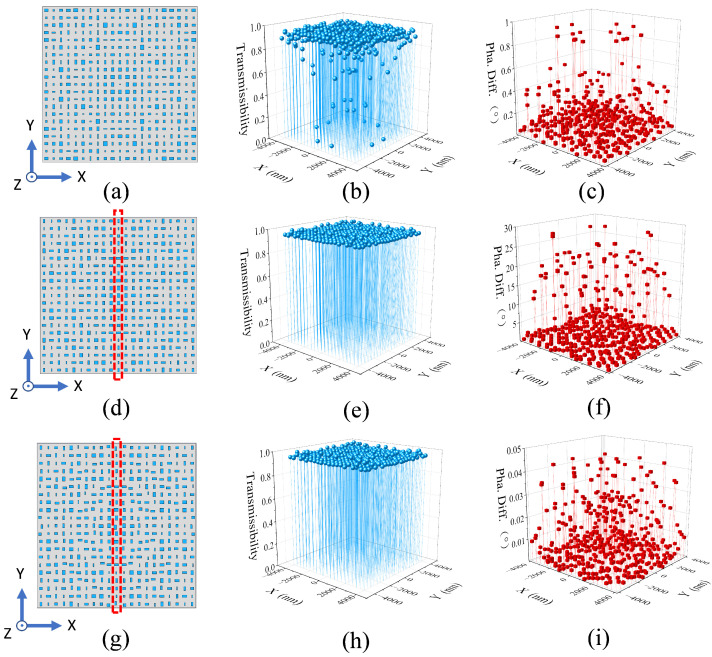
(Color online) Comparison between the typical and threshold-improved metalenses. (**a**–**c**) The top view, nanopillar transmittance, and phase difference of the typical metalens. (**d**–**f**) Corresponding figures of the threshold-improved metalens. (**g**) Top view of the optimized metalens. (**h**) The nanopillar transmittance. (**i**) The phase difference.

In this subsection, we will analyze the effects of only increasing the nanopillar-transmittance threshold. The threshold-improved metalens is built as follows. Abundant nanopillars are simulated, and those with a transmittance of below 0.95 are deleted. Then, the rest of the nanopillars are used to construct the metalens with the common method. As shown in Figure 2, the threshold-improved metalens is compared with the typical metalens. Figure 2a,d shows the top view of the typical and threshold-improved metalens, respectively. Figure 2b,e are the corresponding transmittance diagrams of nanopillars placed on the metalenses. The diagrams clearly show that all nanopillars of the threshold-improved metalens have transmittance of over 0.95, which is much higher and more uniform than that of the typical metalens.

**Figure 3:**
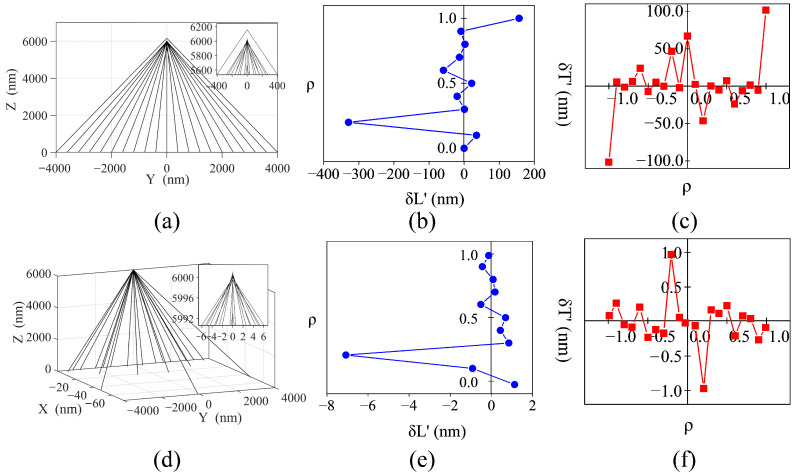
(Color online) The effects of phase correction on the metalens performance. (**a**–**c**) The light tracks, longitudinal and transverse spherical aberrations after single row nanopillars of the threshold-improved metalens. (**d**–**f**) Corresponding results after single row nanopillars of the optimized metalens. The blue and red lines are used to guide the eye.

However, this method abandons a lot of the nanopillars, so the number of phases that the nanopillars can modulate is dramatically reduced. Thus, increasing the nanopillar-transmittance threshold can notably amplify the difference between the desired phase and the nanopillar propagation phase. To illustrate this disadvantage, we compare the phase difference of nanopillars between these two metalenses. According to Equation (4), the phase difference of every nanopillar is calculated, where (x,y) refers to the actual coordinate of the nanopillar placed on the substrate. To make the comparison explicit, Figure 2c,f are plotted with the calculated data. Figure 2c is the phase difference of the traditional metalens, and Figure 3f is that of the threshold-improved metalens. The figures show that the phase difference of the traditional metalens is in the range of 0–1°, but the phase difference of the threshold-improved metalens is amplified to the level of 0–30°. Such a large phase difference will seriously affect the metalens performance.

(4) $$\Delta \varphi =|\varphi_{NPP}\left(x,y\right)-\varphi_{DP}\left(x,y\right)|$$

### 3.2. The Phase Correction

In this subsection, on the basis of improving the transmittance threshold, we will correct the nanopillar phase difference and analyze the effects of phase correction on the metalens performance. As in the previous subsection, only the nanopillars with transmittance of over 0.95 are used to build the metalens. Furthermore, the nanopillar phase difference is corrected to be below 0.05°with the newly proposed method. The relevant results are shown in Figure 2. Compared with the threshold-improved metalens (Figure 2d), the phase correction makes the metalens have an irregular nanopillar distribution (Figure 2g). As seen in Figure 2e,h, the nanopillar transmittance is also high and uniform after the phase correction. On the other hand, Figure 2f shows that the phase difference of the threshold-improved metalens is in the range of 0°–30°. However, Figure 2i presents that the phase difference is well controlled within 0.05°after the threshold-improved metalens has been phase corrected. In this way, the improved and corrected metalens has both high transmittance and low phase difference, which is named “optimized metalens”.

#### 3.2.1. The Effects of Phase Correction on Light Track

To illustrate the advantages of the phase correction, we investigate the light tracks passing through single row nanopillars of the threshold-improved and optimized metalenses, applying the way of calculation (shown as the red dotted box in Figure 2d,g). For these nanopillars, their coordinates (*x*, *y*, 0) of actual placement and propagation phase φ are known. Combining with the focusing phase profile in Equation (1), the coordinates (0, 0, $z_{ip}$) of the intersection point between the light ray and the optical axis can be calculated by Equation (5). Therefore, based on the nanopillar coordinates (*x*, *y*, 0) and the intersection point coordinates (0, 0, $z_{ip}$), the light tracks are obtained and drawn as in Figure 3a,d. For the threshold-improved metalens, Figure 3a shows that the incident light is focused to the level of 5600–6200 nm. The light tracks of the optimized metalens are given in Figure 3d. It is observed that the incident light is focused in the range of 5992–6002 nm, which is improved by about 60 times.

(5) $$z_{ip}=\frac{\pi (x^{2}+y^{2})}{\varphi \lambda }-\frac{\varphi \lambda }{4\pi }$$

#### 3.2.2. The Effects of Phase Correction on Spherical Aberration

Based on the results of the study of the light tracks, the spherical aberration of the metalens is obtained in this paragraph. Spherical aberration is defined as a variation with aperture of the focal length or image distance, which is represented by longitudinal spherical aberration $\delta L^{\prime }$ and transverse spherical aberration $\delta T^{\prime }$, respectively. $\delta L^{\prime }$ and $\delta T^{\prime }$ are expressed as Equations (6) and (7) in this paper, where *f* is the designed focal length (6000 nm), and $U^{\prime }$ is the angle between the light ray and the optical axis. Figure 3b,e shows the longitudinal spherical aberration as a plot of the axial intercept location as a function of the entrance pupil radius ρ. The value of ρ is calculated by Equation (8), where $R_{max}$ is equal to half the length of the metalens. Figure 3c,f present the ray fan plot, which refers to the transverse spherical aberration as a function of entrance pupil radius ρ. In these figures, Figure 3 presents the spherical aberrations of the threshold-improved metalens, and Figure 3e,f show the results of the optimized metalens. According to the results, the performances of the phase-corrected metalens are improved.

(6) $$\delta L^{\prime }=z_{ip}-f$$

(7) $$\delta T^{\prime }=\delta L^{\prime }\tan\left(U^{\prime }\right)$$

(8) $$\rho =\pm \frac{\sqrt{x^{2}+y^{2}}}{R_{max}}$$

### 3.3. Simulation Results

**Figure 4:**
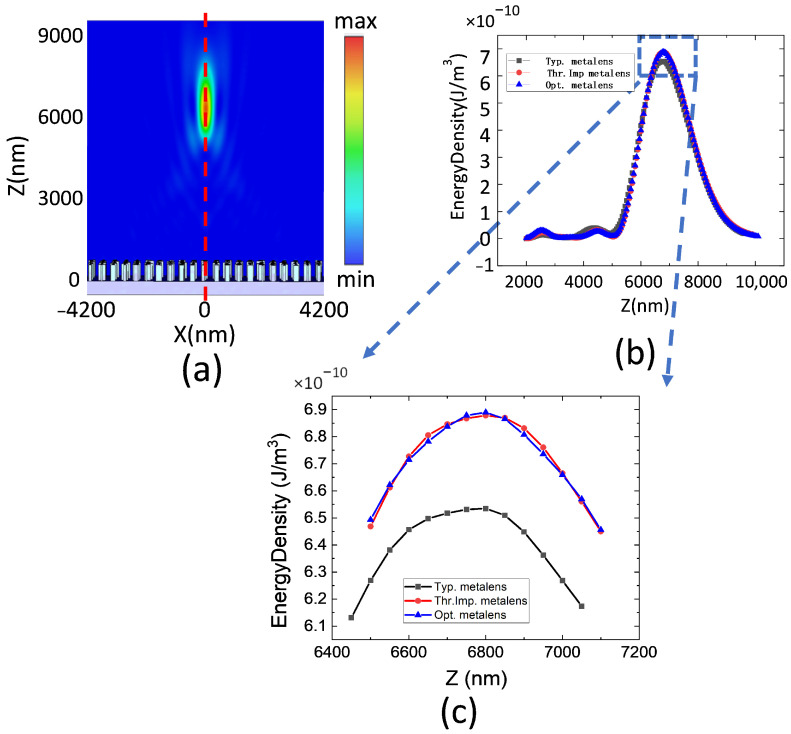
(Color online) The light focusing results of the metalenses. (**a**) The energy density of the focused light for the conventional metalens. (**b**,**c**) The energy density along the optical axis. The lines are used to guide the eye.

After the threshold improvement and phase correction, the optimized metalens with numerical aperture (NA) = 0.57 is obtained, as shown in Figure 2. In order to demonstrate the optimization results, we compared the focusing effects between the conventional, threshold-improved, and optimized metalenses. By means of the finite element simulation, a 532 nm light beam under equal conditions is focused with these metalenses, respectively. The simulated result of the focusing energy density is shown in Figure 4a. To make a clear comparison, the data along the optical axis (the red dashed line in Figure 4) are extracted and drawn as in Figure 4b,c. In the figures, the black squares refer to the data of the traditional metalens, the red circles are those of the threshold-improved metalens, the blue triangles represent those of the optimized metalens, and the energy scale is only a relative value to compare the energy before and after optimization. At other energies, the performance of the optimized metalens will also be well improved. As seen in Figure 4c, the energy densities of the threshold-improved and optimized metalenses are at the same level, and they are much larger than that of the traditional metalens. This indicates that the threshold improvement increases the overall transmittance of the metalens obviously. On the other hand, for the optimized metalens, the energy density distribution around the peak is much more symmetrical than that of the typical and threshold-improved metalenses. These data conform to Gaussian distribution, and the corresponding Gaussian fitting converges. However, the Gaussian fitting of the data about the typical and threshold-improved metalenses does not converge. Furthermore, comparing the results between the threshold-improved and optimized metalenses, the peak value of the optimized metalens is larger than that of the threshold-improved metalens, and the values on both sides are smaller. This indicates that some light rays of the optimized metalens cluster closer to the focal point, which means that the metalens has a smaller spherical aberration. These results prove that the phase correction reduces the spherical aberration of the metalens dramatically. The simulation results are consistent with the analyses in the preceding paragraphs.

## 4. Conclusions

In summary, we have presented a method by optimizing the exact position of each nanopillar. This method improves the nanopillar transmittance and corrects the phase difference in the metasurface. As described above, a metalens is designed and simulated to validate the method. It can be seen that the optimized metalens has higher transmittance, lower phase differences, and smaller spherical aberrations. The method that can successfully solve the two problems mentioned in this paper is an effective way to optimize the metasurface, which can be applied to most metasurface fields: for example, chromatic aberration correction, high NA lenses, holographic imaging, etc. In order to meet the realization of more complex nanostructures, many promising fabrication techniques have been developed and incorporated into the fabrication of metasurfaces. Our method is to make improvements in the design of the metasurface; there is no problem with the fabrication technology. It is expected to be widely used in the development and application of future integrated optical systems.

## Data Availability

The data presented in this study are available on request from the corresponding author.

## References

[1] Jang J.S., Javidi B. (2004). Three-dimensional projection integral imaging using micro-convex-mirror arrays. Opt. Express.

[2] Arai J., Kawai H., Okano F. (2007). Microlens arrays for integral imaging system. Appl. Opt..

[3] Davies N.A., McCormick M., Brewin M. (1994). Design and analysis of an image transfer system using microlens arrays. Opt. Eng..

[4] Chekulaev D., Garber V., Kaplan A. (2013). Free carrier plasma optical response and dynamics in strongly pumped silicon nanopillars. J. Appl. Phys..

[5] Chen H.T., Taylor A.J., Yu N. (2016). A review of metasurfaces: Physics and applications. Rep. Prog. Phys..

[6] Chen W.T., Zhu A.Y., Sanjeev V., Khorasaninejad M., Shi Z., Lee E., Capasso F. (2018). A broadband achromatic metalens for focusing and imaging in the visible. Nat. Nanotechnol..

[7] Engelberg J., Levy U. (2020). The advantages of metalenses over diffractive lenses. Nat. Commun..

[8] Genevet P., Capasso F., Aieta F., Khorasaninejad M., Devlin R. (2017). Recent advances in planar optics: From plasmonic to dielectric metasurfaces. Optica.

[9] Khorasaninejad M., Capasso F. (2017). Metalenses: Versatile multifunctional photonic components. Science.

[10] Khorasaninejad M., Zhu A.Y., Roques-Carmes C., Chen W.T., Oh J., Mishra I., Devlin R.C., Capasso F. (2016). Polarization-Insensitive Metalenses at Visible Wavelengths. Nano Lett..

[11] Hsiao H.H., Chu C.H., Tsai D.P. (2017). Fundamentals and Applications of Metasurfaces. Small Methods.

[12] Khorasaninejad M., Chen W.T., Devlin R.C., Oh J., Zhu A.Y., Capasso F. (2016). Metalenses at visible wavelengths: Diffraction-limited focusing and subwavelength resolution imaging. Science.

[13] Kildishev A.V., Boltasseva A., Shalaev V.M. (2013). Planar Photonics with Metasurfaces. Science.

[14] Wang S., Wu P.C., Su V.C., Lai Y.C., Chen M.K., Kuo H.Y., Chen B.H., Chen Y.H., Huang T.T., Wang J.H. (2018). A broadband achromatic metalens in the visible. Nat. Nanotechnol..

[15] Yu N., Capasso F. (2014). Flat optics with designer metasurfaces. Nat. Mater..

[16] Zheng G., Muehlenbernd H., Kenney M., Li G., Zentgraf T., Zhang S. (2015). Metasurface holograms reaching 80% efficiency. Nat. Nanotechnol..

[17] Chen D., Wang J., Wang S., Zhao S., Qi Y., Sun X. (2021). The bifocal metalenses for independent focusing of orthogonally circularly polarized light. J. Phys. Appl. Phys..

[18] Aieta F., Genevet P., Yu N., Kats M.A., Gaburro Z., Capasso F. (2012). Out-of-Plane Reflection and Refraction of Light by Anisotropic Optical Antenna Metasurfaces with Phase Discontinuities. Nano Lett..

[19] Li Z., Palacios E., Butun S., Aydin K. (2015). Visible-Frequency Metasurfaces for Broadband Anomalous Reflection and High-Efficiency Spectrum Splitting. Nano Lett..

[20] Park J., Kang J.H., Kim S.J., Liu X., Brongersma M.L. (2017). Dynamic Reflection Phase and Polarization Control in Metasurfaces. Nano Lett..

[21] Wong A.M.H., Eleftheriades G.V. (2018). Perfect Anomalous Reflection with a Bipartite Huygens’ Metasurface. Phys. Rev. X.

[22] Zhang X., Tian Z., Yue W., Gu J., Zhang S., Han J., Zhang W. (2013). Broadband Terahertz Wave Deflection Based on C-shape Complex Metamaterials with Phase Discontinuities. Adv. Mater..

[23] Jin L., Dong Z., Mei S., Yu Y.F., Wei Z., Pan Z., Rezaei S.D., Li X., Kuznetsov A.I., Kivshar Y.S. (2018). Noninterleaved Metasurface for (26-1) Spin- and Wavelength-Encoded Holograms. Nano Lett..

[24] Jin L., Huang Y.W., Jin Z., Devlin R., Dong Z., Mei S., Jiang M., Chen W.T., Wei Z., Liu H. (2019). Dielectric multi-momentum meta-transformer in the visible. Nat. Commun..

[25] Chen W.T., Yang K.Y., Wang C.M., Huang Y.W., Sun G., Chiang I.D., Liao C.Y., Hsu W.L., Lin H.T., Sun S. (2014). High-Efficiency Broadband Meta-Hologram with Polarization-Controlled Dual Images. Nano Lett..

[26] Ni X., Kildishev A.V., Shalaev V.M. (2013). Metasurface holograms for visible light. Nat. Commun..

[27] Wen D., Yue F., Li G., Zheng G., Chan K., Chen S., Chen M., Li K.F., Wong P.W.H., Cheah K.W. (2015). Helicity multiplexed broadband metasurface holograms. Nat. Commun..

[28] Mehmood M.Q., Mei S.T., Hussain S., Huang K., Siew S.Y., Zhang L., Zhang T.H., Ling X.H., Liu H., Teng J.H. (2016). Visible-Frequency Metasurface for Structuring and Spatially Multiplexing Optical Vortices. Adv. Mater..

[29] Ming Y., Intaravanne Y., Ahmed H., Kenney M., Lu Y.Q., Chen X.Z. (2022). Creating Composite Vortex Beams with a Single Geometric Metasurface. Adv. Mater..

[30] Yang Y.M., Wang W.Y., Moitra P., Kravchenko I., Briggs D.P., Valentine J. (2014). Dielectric Meta-Reflectarray for Broadband Linear Polarization Conversion and Optical Vortex Generation. Nano Lett..

[31] Arbabi A., Horie Y., Bagheri M., Faraon A. (2015). Dielectric metasurfaces for complete control of phase and polarization with subwavelength spatial resolution and high transmission. Nat. Nanotechnol..

[32] Mueller J.P.B., Rubin N.A., Devlin R.C., Groever B., Capasso F. (2017). Metasurface Polarization Optics: Independent Phase Control of Arbitrary Orthogonal States of Polarization. Phys. Rev. Lett..

[33] Zhao Y., Belkin M.A., Alu A. (2012). Twisted optical metamaterials for planarized ultrathin broadband circular polarizers. Nat. Commun..

[34] Huang K., Dong Z., Mei S., Zhang L., Liu Y., Liu H., Zhu H., Teng J., Luk’yanchuk B., Yang J.K. (2016). Silicon multi-meta-holograms for the broadband visible light. Laser Photonics Rev..

[35] Huang K., Zhao D., Tjiptoharsono F., Chen Y., Wong C.P.Y., Tang X., Yang J.K.W., Dong Z. (2020). Bio-inspired Photonic Masquerade with Perturbative Metasurfaces. ACS Nano.

[36] Decker M., Staude I., Falkner M., Dominguez J., Neshev D.N., Brener I., Pertsch T., Kivshar Y.S. (2015). High-Efficiency Dielectric Huygens’ Surfaces. Adv. Opt. Mater..

[37] Staude I., Miroshnichenko A.E., Decker M., Fofang N.T., Liu S., Gonzales E., Dominguez J., Luk T.S., Neshev D.N., Brener I. (2013). Tailoring Directional Scattering through Magnetic and Electric Resonances in Subwavelength Silicon Nanodisks. ACS Nano.

[38] Yu Y.F., Zhu A.Y., Paniagua-Dominguez R., Fu Y.H., Luk’yanchuk B., Kuznetsov A.I. (2015). High-transmission dielectric metasurface with 2 phase control at visible wavelengths. Laser Photonics Rev..

[39] Chen D., Wang J., Qi Y., Wang S., Xue Q., Sun X. (2020). Polarization-insensitive dielectric metalenses with different numerical apertures and off-axis focusing characteristics. J. Opt. Soc. Am. B Opt. Phys..

[40] Engelberg J., Zhou C., Mazurski N., Bar-David J., Kristensen A., Levy U. (2020). Near-IR wide-field-of-view Huygens metalens for outdoor imaging applications. Nanophotonics.

[41] Zheng C., Li J., Yue Z., Li J., Liu J., Wang G., Wang S., Zhang Y., Zhang Y., Yao J. (2022). All-Dielectric Trifunctional Metasurface Capable of Independent Amplitude and Phase Modulation. Laser Photonics Rev..

[42] Liu Y., Chen L., Zhou C., Guo K., Wang X., Hong Y., Yang X., Wei Z., Liu H. (2022). Theoretical Study on Generation of Multidimensional Focused and Vector Vortex Beams via All-Dielectric Spin-Multiplexed Metasurface. Nanomaterials.

[43] Ma Z., Hanham S.M., Albella P., Ng B., Lu H.T., Gong Y., Maier S.A., Hong M. (2016). Terahertz All-Dielectric Magnetic Mirror Metasurfaces. ACS Photonics.

[44] Hong X., Feng S., Guo H., Li C. (2020). A beam deflector with dielectric metasurfaces in the terahertz region. Laser Phys..

[45] Arbabi A., Horie Y., Ball A.J., Bagheri M., Faraon A. (2015). Subwavelength-thick lenses with high numerical apertures and large efficiency based on high-contrast transmitarrays. Nat. Commun..

[46] Zhang L., Ding J., Zheng H., An S., Lin H., Zheng B., Du Q., Yin G., Michon J., Zhang Y. (2018). Ultra-thin high-efficiency mid-infrared transmissive Huygens meta-optics. Nat. Commun..

[47] Shalaev M.I., Sun J., Tsukernik A., Pandey A., Nikolskiy K., Litchinitser N.M. (2015). High-Efficiency All-Dielectric Metasurfaces for Ultracompact Beam Manipulation in Transmission Mode. Nano Lett..

[48] Chen M.H., Yen C.W., Guo C.C., Su V.C., Kuan C.H., Lin H.Y. (2021). Polarization-insensitive GaN metalenses at visible wavelengths. Sci. Rep..

[49] Xie J., Guo H., Zhuang S., Hu J. (2021). Polarization-controllable perfect vortex beam by dielectric metasurface. Opt. Express.

